# DsTRD: Danshen Transcriptional Resource Database

**DOI:** 10.1371/journal.pone.0149747

**Published:** 2016-02-24

**Authors:** Yuxuan Shao, Jiabo Wei, Fangli Wu, Haihua Zhang, Dongfeng Yang, Zongsuo Liang, Weibo Jin

**Affiliations:** Institute of Bioengineering, College of Life Sciences, Zhejiang Sci-Tech University, Hangzhou, 310018, China; Huazhong University of Science and Technology, CHINA

## Abstract

*Salvia miltiorrhiza* has been comprehensively studied as a medicinal model plant. However, research progress on this species is significantly hindered by its unavailable genome sequences and limited number of expressed sequence tags in the National Center for Biotechnology Information database. Thus, a transcript database must be developed to assist researchers to browse, search, and align sequences for gene cloning and functional analysis in *S*. *miltiorrhiza*. In this study, the Danshen Transcriptional Resource Database (DsTRD) was built using 76,531 transcribed sequences assembled from 12 RNA-Seq transcriptomes. Among these 12 RNA-seq data, ten were downloaded from NCBI database. The remaining two were enced on the Hiseq2000 platform using the stem and hairy-root of *S*. *miltiorrhiza*. The transcripts were annotated as protein-coding RNAs, long non-coding RNAs, microRNA precursors, and phased secondary small-interfering RNA genes through several bioinformatics methods. The tissue expression levels for each transcript were also calculated and presented in terms of RNA-Seq data. Overall, DsTRD facilitates browsing and searching for sequences and functional annotations of *S*. *miltiorrhiza*. DsTRD is freely available at http://bi.sky.zstu.edu.cn/DsTRD/home.php.

## Introduction

*Salvia miltiorrhiza* Bunge is a perennial plant that belongs to the Lamiaceae family. The dried root or rhizome of this plant is called “Danshen” in traditional Chinese medicine. *S*. *miltiorrhiza* is comprehensively studied as a medicinal model plant because of its small genome, short life cycle, and stable genetic transformation system. Elucidation and regulation of biosynthesis pathways involving the active ingredients of *S*. *miltiorrhiza* are considered major research topics. However, the unavailable genome sequence and limited nucleotide sequences (only 1,048 nucleotides and 10,288 expressed sequence tags, ESTs) of *S*. *miltiorrhiza* in the National Center for Biotechnology Information (NCBI) database significantly hinder research progress on this species, specifically in terms of molecular growth mechanisms, developmental and stress responses, and biosynthesis of active ingredients.

Numerous transcriptomes of *S*. *miltiorrhiza* have been extensively recovered through high-throughput sequencing and deposited into the NCBI Sequence Read Archive (SRA) database. Nevertheless, researchers should continuously analyze large amounts of raw sequencing reads. Despite the currently available routine processes and methods, considerable time and effort are expended to retrieve sequences and features and install diverse software packages. Thus, a transcript resource, which can assemble and annotate raw reads and can be easily searched by humans and computers, must be developed. In this regard, we developed the Danshen Transcriptional Resource Database (DsTRD), which is a simple but comprehensive transcript resource for *S*. *miltiorrhizae*.

## Materials and Methods

### RNA-Seq data information

Ten RNA-seq data including 7 pair-end sequencing data based on Hiseq2000 platform and three single-end sequencing data based on 454 platform were downloaded from NCBI database with Sequence Read Archive (SRA) formation (S1 Table).

In addition, two RNA-seq data (RNAseq_1 and RNAseq_2) were added for transcripts assambly and tissue expression analysis by using the stem and hairy-root of *S*. *miltiorrhiza* and sequencing on the Hiseq2000 platform (S1 Table). The stems of *S*. *miltiorrhiza* were collected from a Salvia planting base in shangluo, China. And the hairy-root of *S*. *miltiorrhiza* were cultured by our lab.

### Transcriptional assembly annotation and quantification

**Fig 1:**
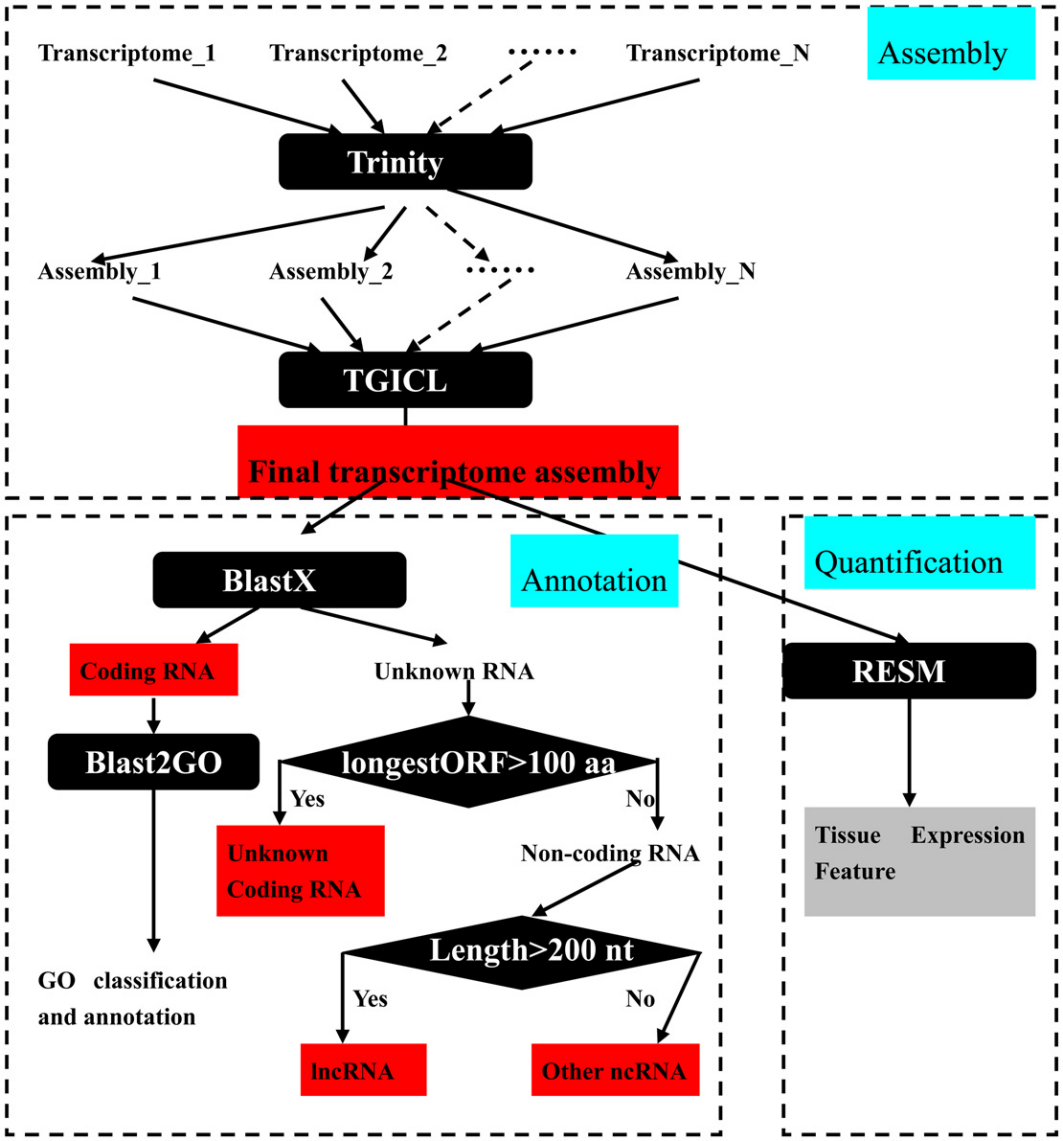
Flowchart of the assembly, annotation, and quantification of RNA-seq in *S*. *miltiorrhiza*.

The assembly flowchart of *S*. *miltiorrhiza* RNA-Seq data is shown in Fig 1. The adapters and poor quality sequences were firstly removed from RNA-seq data using FASTX toolkit downloaded from http://hannonlab.cshl.edu/fastx_toolkit/download.html. Clean sequencing data were assembled into transcripts by using Trinity software with the parameters “—min_kmer_cov 3—min_glue 3” [1]. All assembled transcripts from the total samples were clustered by tgicl v2.1 with default parameters [2] (S2 Table). These RNAs were annotated and classified by the Blast2GO software (http://www.blast2go.com/) with the NR database and e-values < 1 e^−5^. Unknown transcripts were classified as unknown coding RNA or noncoding RNA (ncRNA) based on the length of the longest open reading frame (> 100 amino acids = unknown coding RNA, < 100 amino acids = ncRNA). Finally, ncRNAs were considered as long ncRNAs (lncRNAs) if their length is > 200 nt. The remaining ncRNAs were considered as other ncRNAs. To profile the tissue expression features of the assembled transcripts, we derived the count and expression levels (fragments per kilobase per million mapped reads) from RNA-seq data by using the RSEM pipeline with default parameters [3]. RNA-seq datasets were selected from the 12 RNA-seq datasets (S1 Table). The selected sets were produced from the same sequencing platform (Hiseq2000) with pair-end sequencing, high-depth sequencing, and five different tissue samples (SRR1043998, SRR1045051, SRR1020591, RNAseq_1, and RNAseq_2) (S3 Table).

### Annotation and quantification of miRNAs in *S*. *miltiorrhiza*

Four sRNA-Seq data were downloaded from the NCBI database with SRA formation (S3 Table). The other sRNA-Seq data (sRNAseq_1) were added for miRNA identification and tissue expression analysis; these data were sequenced on the Hiseq2000 platform by using the hairy root of *S*. *miltiorrhiza* (S3 Table).

In DsTRD, miRNAs were classified into three types. The first type is named as “miRxxx-like,” with a sequence similar to the known “miRxxx” in other plants (published in miRBase with the version 21.0). The precursor with stem-loop structure of this miRNA could not be retrieved from the assembled transcripts. The second type is named as “Smi-miRxxx,” with a sequence also similar to the known “miRxxx” in other plants (published in miRBase with the version 21.0). But the precursor with stem-loop structure of this miRNA could be retrieved from the assembled transcripts. The third is novel miRNA identified in a plant and satisfied the criteria established by Meyers et al. [4] and Thakur et al [5]. Among novel miRNAs, those supported with the reads of miRNA* were annotated as real novel miRNAs, whereas the unsupported reads were annotated as novel miRNA candidates. To profile the tissue expression features of miRNAs, we derived the counts and expression levels (reads per million, RPM) from sRNA-seq data by using an in-house Perl script.

### Annotation and quantification of the phased secondary small interfering RNAs (phasiRNA) in *S*. *miltiorrhiza*

Phased, secondary small-interfering RNAs (phasiRNAs) are important in post-transcriptional regulatory networks in plants [6]. Well-characterized trans-acting siRNAs (tasiRNAs), as a special subgroup of phasiRNAs, are initiated by miRNA-mediated cleavage and converted to dsRNA to yield siRNAs in a 21-nt phase [6–8]. All sRNA-Seq data applied in the miRNA annotation were used for phasiRNA identification and quantification through the PhaseTank pipeline with default parameters [9]. The identified phasiRNA genes triggered by miRNAs were also considered as tasiRNAs and named “TASxxx,” whereas the remaining phasiRNA genes were named “PASxxx” in the DsTRD. We also derived the counts and RPM expression levels to profile the tissue expression features of phasiRNAs by using an in-house Perl script.

### Database design and implementation

The web pages of DsTRD were constructed using a Hypertext Preprocessor (PHP) language and run on a Linux system (Centos 6.4). All data were stored in a MySQL (5.1.66) database (http://www.mysql.com), whereas the assembled transcripts, miRNAs, and phasiRNAs for download were stored as files in FASTA format.

## Results and Discussion

### Web interface of DsTRD

**Fig 2:**
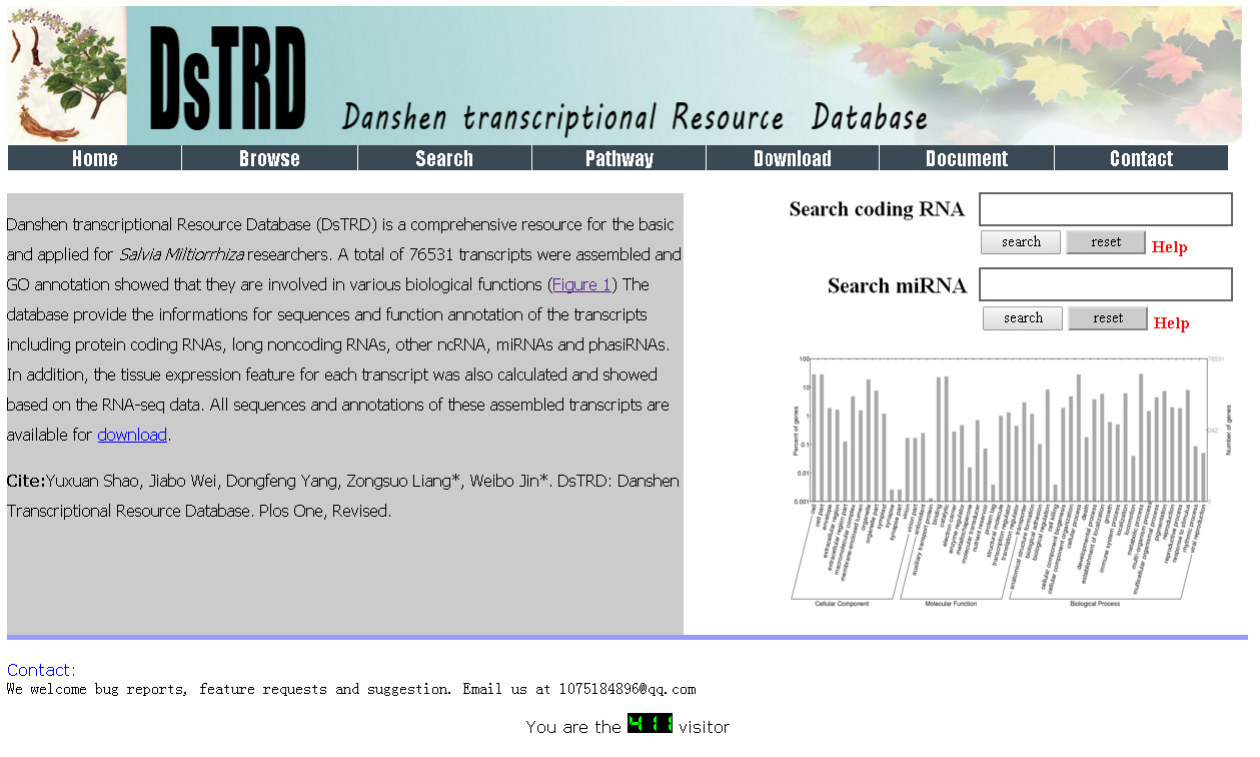
DsTRD home page.

We created a freely accessible web interface for DsTRD. The interface comprises six core-page sections: “Home,” “Browse,” “Search,” “Pathway,” and “Download” (Fig 2).

### Home page

Users can access DsTRD at http://bi.sky.zstu.edu.cn/DsTRD/home.php. The home page contains an introduction to DsTRD and a search engineer (Fig 2). This section provides users an outline of DsTRD and can be conveniently used to search gene information.

## Browse

**Fig 3:**
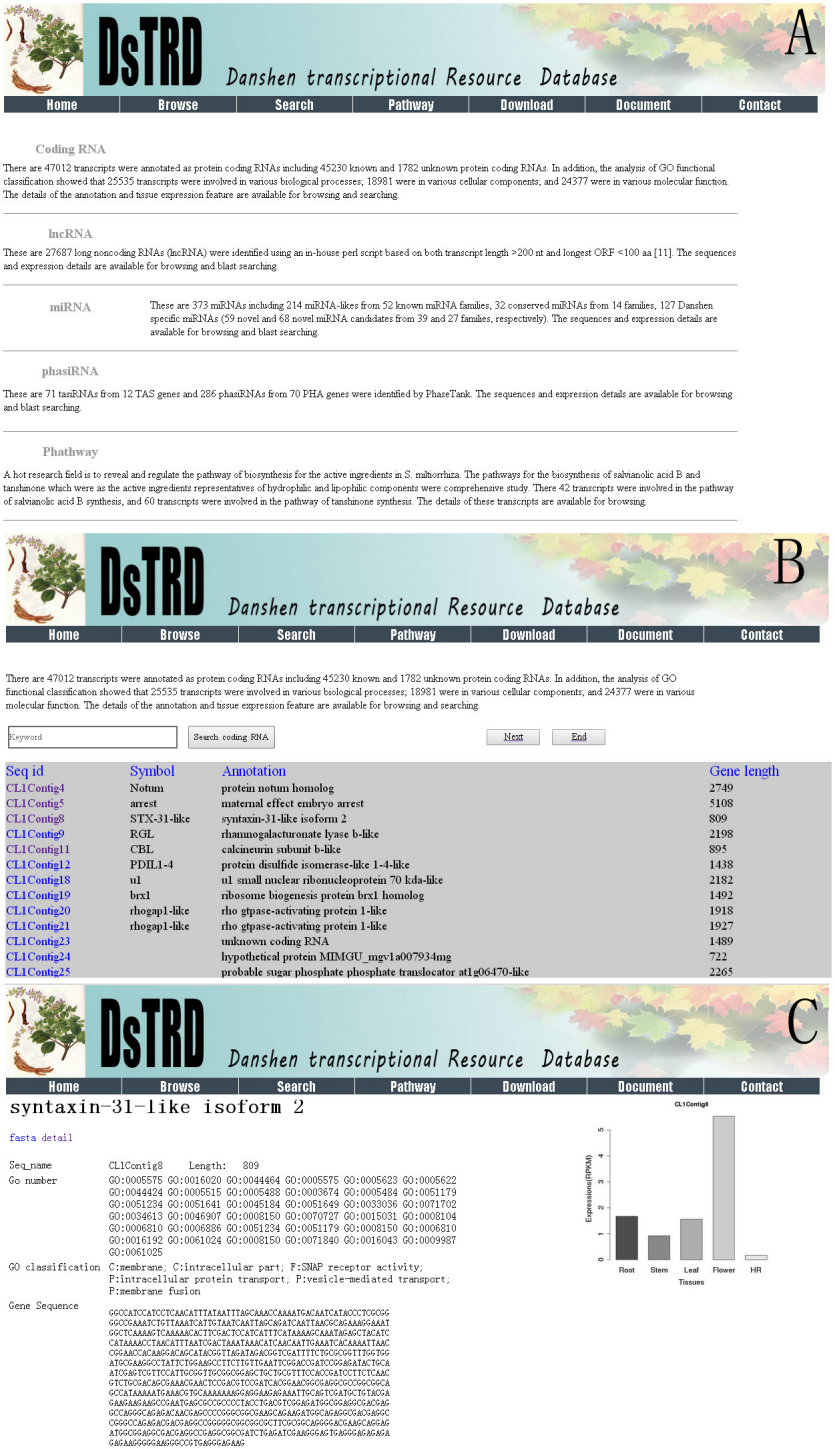
Information on various RNAs. (A) Users can browse the summaries of four kinds of RNAs. (B) Users can search and view the features of coding RNA. (C) Information page of RNA.

Browse facilitates users to browse the details of various transcripts, including coding RNAs, lncRNAs, miRNAs, and phasiRNAs (Fig 3A). Users can click the coding RNA, lncRNA, miRNA, and phasiRNA to browse the summaries (Seq id, annotation, and the length of the transcripts) of the corresponding RNAs. Moreover, data source can be selected in the search results to download the sequences and information in FASTA format (Fig 3B). The details of each transcript is displayed by clicking on “Seq id.” The tissue expression features for each transcript is also shown in the information page (Fig 3C).

## Search

**Fig 4:**
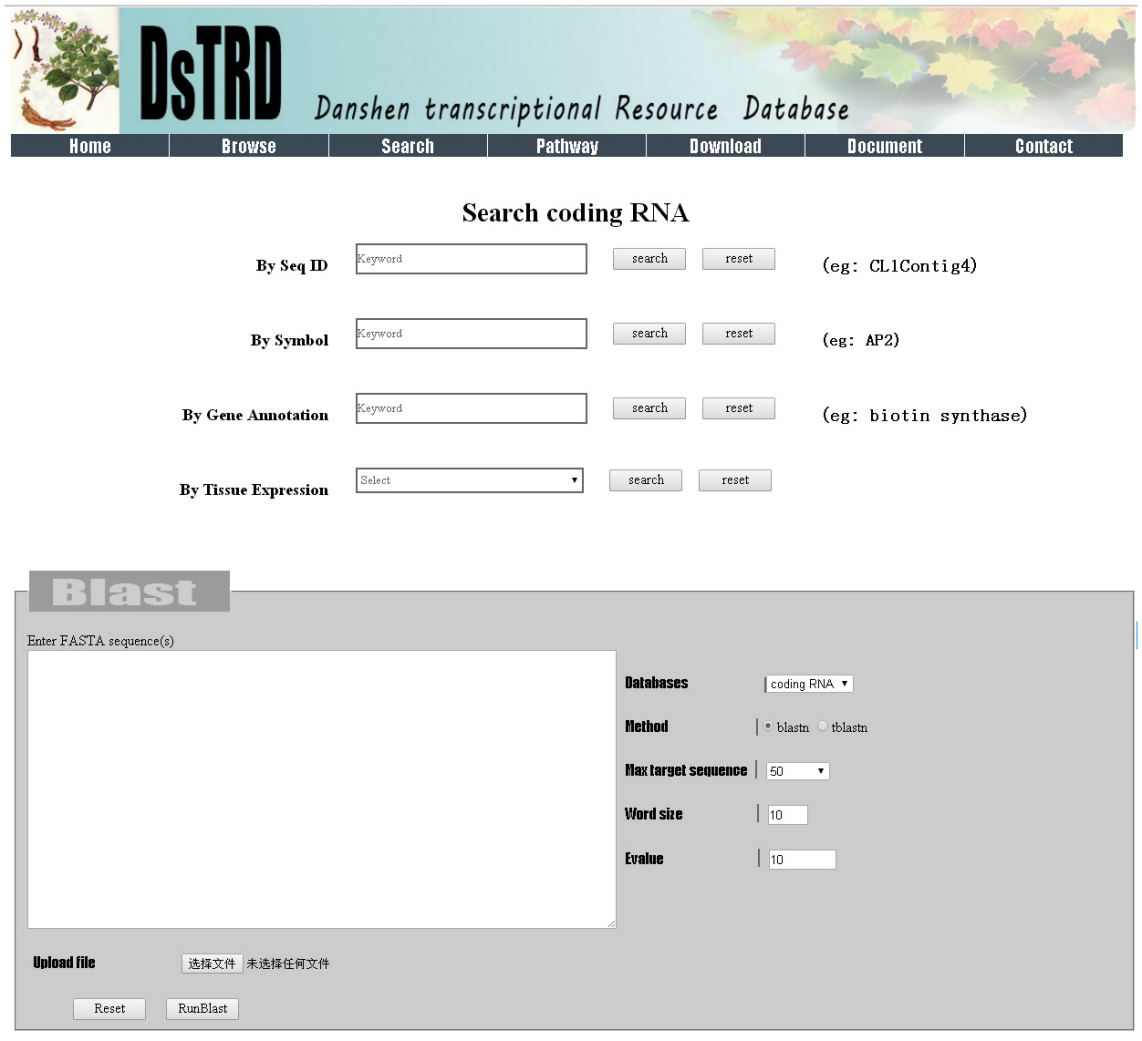
Search and Blast page.

Search functionalities are designed to facilitate users to retrieve useful information. In the search frame (Fig 4), users can search for information regarding id, gene name, gene annotation, gene ontology (GO) number, and GO classification by encoding one or more key words. As DsTRD maintained the nucleotide-sequence data of various RNAs, a sequence alignment tool, namely, the Basic Local Alignment Search Tool (BLAST), was integrated into DsTRD. Users can compare a query sequence with a library of coding RNA, lncRNA, miRNA, and phasiRNA sequences by using BLAST search and identify the sequences that closely resemble the query sequence. BLAST will meet the users’ requirements in finding homologous transcripts of interest. In the BLAST frame (Fig 4), users can supply one or more query sequences by uploading or directly pasting these sequences to search against the available databases using BLAST default parameters. Users can also specify additional parameters for BLAST search to control search sensitivity and result format as desired. The BLAST results are displayed in another page in a pairwise format by default.

## Pathway

**Fig 5:**
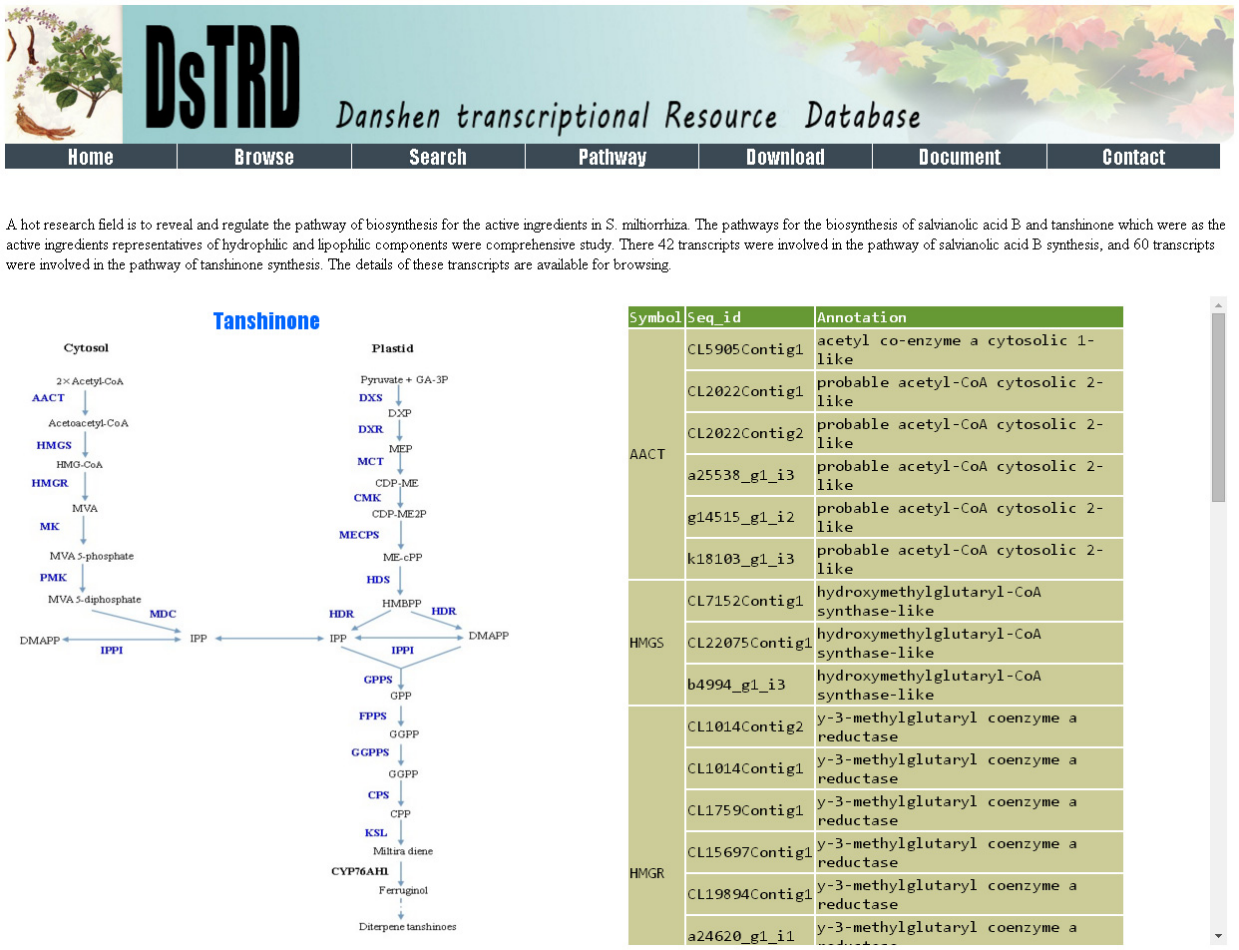
Pathway page.

A hot research field is included to reveal and regulate biosynthesis pathways involving the active ingredients of *S*. *miltiorrhiza*. The biosynthesis pathways for salvianolic acid B and tanshinone, which are active ingredient representatives of the hydrophilic and lipophilic components, respectively, were comprehensively studied. By searching DsTRD, we found that 46 transcripts participate in salvianolic acid B synthesis, and 60 transcripts are involved in tanshinone synthesis (Fig 5). In the pathway page, users can view the outlines of these two pathways. Users can also view detailed information on each RNA of the enzyme by clicking the enzyme name (showed in blue font) on the pathways.

## Downloads

DsTRD data can be downloaded to perform local analysis. In the download page, users can download coding RNAs, lncRNAs, miRNAs and their precursors, and phasiRNA and their precursors in FASTA format.

## Conclusion

*S*. *miltiorrhiza* is an extensively investigated medicinal model plant because of its small genome, short life cycle, and stable genetic transformation system. However, the lack of available genome sequences and the limited EST sequences stored in NCBI for *S*. *miltiorrhiza* considerably restrict study progress on the molecular mechanisms of this species. In this study, we aim to produce large numbers of transcript sequences with corresponding annotation information and make these data freely accessible to users. As such, we developed DsTRD, which exhibits simplicity of use for researchers and contains 76531 transcribed sequences assembled from the RNA-seq data. This database provides information regarding the sequences and functional annotations of the transcripts, including protein-coding RNAs, lncRNAs, other ncRNA, miRNAs, and phasiRNAs. The database also includes the tissue expression feature for each transcript, which was calculated and shown based on RNA-seq data. As an efficient bioinformatics tool, DsTRD is important in studying various kinds of molecular processes in *S*. *miltiorrhiza*.

## Availability and Requirements

DsTRD is publicly available at http://bi.sky.zstu.edu.cn/DsTRD/home.php. DsTRD supports all the latest major web browsers, preferably Mozilla Firefox, Google Chrome, or Apple Safari, for visualization and performance purposes.

## Supporting Information

S1 Table Transcriptome data downloaded from the NCBI database according to the accession numbers. (DOC)

S2 Table All assembled transcripts in fasta format. (ZIP)

S3 Table sRNA-Seq data downloaded from the NCBI database according to the accession numbers. (DOC)

## References

[1] Haas BJ, Papanicolaou A, Yassour M, Grabherr M, Blood PD, Bowden J, et al (2013) De novo transcript sequence reconstruction from RNA-seq using the Trinity platform for reference generation and analysis, Nat Protoc, 8, 1494–1512.] 10.1038/nprot.2013.084 23845962 PMC3875132

[2] Pertea G, Huang X, Liang F, Antonescu V, Sultana R, Karamycheva S, et al (2003) TIGR Gene Indices clustering tools (TGICL): a software system for fast clustering of large EST datasets, Bioinformatics, 19, 651–652. 12651724 10.1093/bioinformatics/btg034

[3] Li B and Dewey CN (2011) RSEM: accurate transcript quantification from RNA-Seq data with or without a reference genome, BMC Bioinformatics, 12, 323 10.1186/1471-2105-12-323 21816040 PMC3163565

[4] Meyers BC, Axtell MJ, Bartel B, Bartel DP, Baulcombe D, Bowman JL, et al (2008) Criteria for annotation of plant microRNAs. Plant Cell, 20:3186–3190. 10.1105/tpc.108.064311 19074682 PMC2630443

[5] Thakur V, Wanchana S, Xu M, Bruskiewich R, Quick WP, Mosig A, Zhu XG (2011) Characterization of statistical features for plant microRNA prediction. BMC Genomics, 12: 108 10.1186/1471-2164-12-108 21324149 PMC3053258

[6] Fei Q, Xia R and Meyers BC. (2013) Phased, secondary, small interfering RNAs in posttranscriptional regulatory networks, The Plant cell, 25, 2400–2415. 10.1105/tpc.113.114652 23881411 PMC3753373

[7] Howell MD, Fahlgren N, Chapman EJ, Cumbie JS, Sullivan CM, Givan SA, et al (2007) Genome-wide analysis of the RNA-DEPENDENT RNA POLYMERASE6/DICER-LIKE4 pathway in Arabidopsis reveals dependency on miRNA- and tasiRNA-directed targeting, The Plant cell, 19, 926–942. 17400893 10.1105/tpc.107.050062 PMC1867363

[8] Axtell MJ. (2013) ShortStack: comprehensive annotation and quantification of small RNA genes. RNA, 19, 740–751. 10.1261/rna.035279.112 23610128 PMC3683909

[9] Guo QL, Qu Xiongfei, Jin Weibo. (2015) PhaseTank: genome-wide computational identification of phasiRNAs and their regulatory cascades. Bioinformatics, 31: 284–286. 10.1093/bioinformatics/btu628 25246430

