# Supplementary material for: DsTRD: Danshen Transcriptional Resource Database
Source: PLoS One. 2016 Feb 24;11(2):e0149747. doi: 10.1371/journal.pone.0149747 (PMC4765898; doi:10.1371/journal.pone.0149747)
Supplement: S1 Table — (DOC) [file pone.0149747.s001.doc]

| Accession/No. | Method | Pair/single | tissue |
| --- | --- | --- | --- |
| SRR1043998 | HiSeq 2000 | Pair | root |
| SRR1045051 | HiSeq 2000 | Pair | leaf |
| SRR1020591 | HiSeq 2000 | Pair | flower |
| RNAseq_1 | HiSeq 2000 | Pair | Stem |
| RNAseq_2 | HiSeq 2000 | Pair | Hairy root |
| SRR946948 | HiSeq 2000 | Pair | leaf |
| SRR946949 | HiSeq 2000 | Pair | leaf |
| SRR946950 | HiSeq 2000 | Pair | leaf |
| SRR946951 | HiSeq 2000 | Pair | leaf |
| SRR037804 | 454 GS FLX | Single | root |
| SRR1005880 | 454 GS FLX | Single | leaf+root |
| SRR924322 | 454 GS FLX | Single | hairy root |

Table S1. Transcriptome data downloaded from the NCBI database according to the accession numbers
