# Supplementary material for: DsTRD: Danshen Transcriptional Resource Database
Source: PLoS One. 2016 Feb 24;11(2):e0149747. doi: 10.1371/journal.pone.0149747 (PMC4765898; doi:10.1371/journal.pone.0149747)
Supplement: S3 Table — (DOC) [file pone.0149747.s003.doc]

S3 Table sRNA-Seq data downloaded from the NCBI database according to the accession numbers

| Accession | Method | Tissue |
| --- | --- | --- |
| SRR1557860 | HiSeq 2000 | Root |
| SRR1557861 | HiSeq 2000 | Stem |
| SRR1557862 | HiSeq 2000 | leaf |
| SRR1557863 | HiSeq 2000 | flower |
| sRNAseq_1 | HiSeq 2000 | hairy root |
